# Editorial: Controlled self-assembly and functionalization

**DOI:** 10.3389/fbioe.2022.1016679

**Published:** 2022-09-08

**Authors:** Xing Wang, Yunlong Wu, Zibiao Li, Yanyu Yang

**Affiliations:** ^1^ Beijing National Laboratory for Molecular Sciences, Institute of Chemistry, Chinese Academy of Sciences, Beijing, China; ^2^ University of Chinese Academy of Sciences, Beijing, China; ^3^ Institute of Materials Research and Engineering, A*STAR (Agency for Science Technology and Research), Singapore, Singapore; ^4^ Fujian Provincial Key Laboratory of Innovative Drug Target Research, School of Pharmaceutical Sciences, Xiamen University, Xiamen, China; ^5^ College of Materials Science and Engineering, Zhengzhou University, Zhengzhou, China

**Keywords:** molecular self-assembly, functional biomaterials, drug delivery, biofunctionalization, controllable materials with programmable functions, regenerative medicine

Nature uses a self-assembling hierarchy to construct a hybrid structure of inorganic and organic primitives. Numerous examples in nature graphically illustrate the mechanism of hierarchical self-assembly for building a variety of structurally biomimetic architectures and functions, where multiple components are configured through a step-by-step process by the complex coordinated interactions. In recent years, a library of attractive precursors is being generated and expanding by architectural self-assembly, which provides more opportunities in adjusting and optimizing the physicochemical and properties biological features for target bio-applications, such as drug delivery, tissue engineering, clinical diagnosis, and many others. Thus, amalgamating the physical/chemistry of controlled self-assembly along with biomaterials science will efficiently produce innovative biomaterials from the laboratory to the clinic.

In this Research Topic, five manuscripts by 20 authors containing three review articles and two original research articles were included in the cutting-edge fields of synthesis and characterization of energetic materials, self-assembly and applications as well as the advanced experimental techniques. Review articles mainly presented three aspects of carrier-free nanodrug delivery system, phototherapy and phototherapy-based multimodal synergistic therapy and ceramic toughening strategy by flexibly structural fabrication and functional organization for various biomedical applications. For example, Jiang et al. provided a recent overview on several carrier-free nanodrugs (prodrugs, pure drugs, or amphiphilic drug-drug conjugates) delivery systems. In views of phytochemical self-assembled nanodrugs with many advantages of low toxicity, side effects, high drug loading rate, good pharmacokinetics, stimulus-responsive abilities and synergistic treatment effect, the authors focused on the synthesis, modification and antitumor therapy of these carrier-free nanodrugs based on the natural products as therapeutic agents with different antitumor mechanisms. He et al. summarized the types, structural features, properties, and application of vacancy defects in phototherapeutic nanomaterials, and discuss the significant influence and role of vacancy defects on phototherapy and multimodal synergistic phototherapy. Moreover, as a kind of biomedical used-ceramic material, it should be strictly supervised in clinic because the catastrophic facture can directly result in the medical malpractice and patient pain. Therefore, advances in systematic studies of ceramic toughness and modulation strategies are urgently needed. In this case, Bai et al. summarized several toughening strategies and mechanisms such as reinforcing second phase, surface modification and manufacturing process optimization for achieving the extrinsic toughening for biomedical ceramic. For the two original research articles, the self-assembled nanoparticles were also focused on for the antitumor applications. Because of the urgent need to release the payload quickly in response to inherent biological signal changes, Wang et al. prepared a redox-sensitive PEGylated doxorubicin of PEG-disulfide-DOX prodrug and self-assembled into the biodegradable nanoparticles in aqueous solutions. Taking consideration into the remarkable difference in the extracellular oxidative environment and the reduction potential of the intracellular reducing solution, these prodrug nanoparticles were localized intracellular and stimulated to rapidly degrade to release the drugs once exposure into the targeted tumors. These reduction-sensitive micelles further physically encapsulated the free drugs into polymer carriers and exhibited a two-phase programmed drug release behavior, showing great potential for the development of the advanced payload carriers for effective tumor chemotherapy. In addition, Lin et al. designed and exploited a series of branching polymers for investigating the drug release kinetics using an injectable thermogel. During the gelling process, these highly-branched polymers were encased with each other inefficiently, which could affect their physical properties and stability against gel erosion, thus resulting in a faster release of encapsulated small hydrophobic drugs and proteins.

Therefore, the ambitious goal of this research topic is to underpin the importance of controllable self-assembly, functionality and biomedical applications, which have unique characteristics that enable them to obtain elaborate design and advanced assembly in various bio-applications, and to realize their respective potential in the real world. We sincerely hope to achieve multifunctional biomaterial breakthroughs and process technique innovations in an advanced level that will benefit humanity and enjoy reading in this special edition.

